# Cognition in Patients With Memory Difficulties and Dementia Relative to APOE e4 Status

**DOI:** 10.3389/fpsyg.2021.686036

**Published:** 2021-06-14

**Authors:** Knut Hestad, Knut Engedal, Peter Horndalsveen, Bjørn Heine Strand

**Affiliations:** ^1^Department of Health and Nursing Science, Faculty of Health and Social Sciences, Inland Norway University of Applied Sciences, Elverum, Norway; ^2^Department of Research, Innlandet Hospital Trust, Ottestad, Norway; ^3^Norwegian National Advisory Unit on Ageing and Health, Vestfold County Hospital Trust, Tønsberg, Norway; ^4^Department of Geriatric Medicine, Oslo University Hospital, Oslo, Norway; ^5^Department of Old Age Psychiatry, Innlandet Hospital Trust, Ottestad, Norway; ^6^Department of Chronic Diseases and Ageing, Norwegian Institute of Public Health, Oslo, Norway

**Keywords:** cognition, memory difficulties, minor neurocognitive disorders, major neurocognitive disorders, dementia, APOE e4 status

## Abstract

The aim of this study was to investigate whether cognitive performance was equally influenced by Apolipoprotein E (APOE, with its three alleles, e2, e3, and e4) in patients with subjective cognitive decline (SCD), mild cognitive impairment (MCI), and Alzheimer’s disease (AD). In addition, we examined a group of patients with a combination of Vascular dementia (VaD) and AD (VaD/AD). We asked if the APOE e4 allele influenced cognition in these patient groups in the same way. Our study comprised data from 1,991 patients (55% women), with a mean age of 70.9 years (SD 10.8) and 12.1 years of education (SD 3.8). Of them, 1,111 (56%) had at least one APOE e4 allele; 871 (44%) had one and 240 (12%) had two e4 alleles. Three neurocognitive tests were used to measure cognition: the Mini Mental State Examination (MMSE), the 10-word test of the Consortium to Establish a Registry for Alzheimer’s Disease Word List (CERAD-WL) (immediate and delayed recall), and the Trail Making Test Part A (TMTA). The APOE genotypes were regressed against cognitive function using linear regression, adjusting for diagnosis, age, sex, and education. The interaction diagnosis^∗^APOE was investigated. The allele type had the largest effect on cognitive performance assessed by the CERAD-WL delayed recall test, less for the other tests. Those without the e4 type scored 0.7 units better than those with e4 allele(s) (*p* < 0.001). Furthermore, there was a significant inverse dose-response pattern between number of e4 alleles and cognitive performance; those with one allele scored 0.4 units better than those with two alleles (*p* = 0.006), and those without e4 scored 0.7 units better than those with one e4 (*p* < 0.001). This pattern did not differ between the four diagnostic groups studied.

## Introduction

The human Apolipoprotein E (APOE) gene has three common allelic variants—e*2*, e*3*, and e*4*—which are encoded on chromosome 19 (Mahley et al., 2009). Depending on the genetic variability, the APOE gene has been ascribed significant positive and negative health effects such as longevity and shortened lifespan, neurological and psychosomatic disorders, cognitive decline and Alzheimer disease, altered lipoprotein profile, atherosclerosis and cardiovascular disease, type II diabetes, changes in the immune response, oxidative stress, quality of life, physical activity, and obesity (Spinney, 2014; Klimentidis et al., 2018; Abondio et al., 2019; Kulminski et al., 2019). The e4 allele has been ascribed poor health outcomes and the e2 has been associated with health-promoting effects (Farlow et al., 2004; Fan et al., 2006; Julian et al., 2009; Chou, 2010; Suri et al., 2013; Spinney, 2014; Kulminski et al., 2016; Abondio et al., 2019). According to a review of several studies, it is well known that persons with the e4 allele have an increased risk of developing Alzheimer’s disease (AD) compared with persons without the e4 allele (Saunders et al., 1993). This finding could be secondary to a dosage effect, as persons with homozygote combinations of APOE e4 (e4/e4) have the highest risk for development and early onset of dementia due to AD (Verghese et al., 2011). The clinical hallmark of AD is, among other factors, a progressive decline in episodic memory (McKhann et al., 2011), and a substantial number of studies have reported that the e4 allele contributes significantly to this progressive decline (El Haj et al., 2016). Even though there seems to be a dose-dependent risk regarding the development and course of the second most common dementia type, Vascular dementia (VaD), with a higher risk related to homozygote e4 alleles, more studies are needed to understand how VaD and Apo E status are related to each other (Verghese et al., 2011).

In addition, studies have shown that age-related memory decline in healthy individuals differs between e4 carriers and non-carriers (Caselli et al., 2009). For carriers of e4, decline started before age 60 and accelerated faster than in non-carriers. Those with homozygotic e4 alleles demonstrated the strongest acceleration (Caselli et al., 2009).

Various theories exist to explain how the APOE e4 allele affects the brain. One theory is that the different APOE e variants have various effects on amyloid-β accumulation (cerebral amyloid angiopathy and fibrinogen deposits) in the brain (Hultman et al., 2013). It is further suggested that the e4 variant increases blood-brain-barrier damage (Zipser et al., 2007). Another theory is that the various APOE alleles cause differences in binding and clearance of Aβ (Bell et al., 2007; Deane et al., 2008).

As the e4 allele is reported to have a negative effect on episodic memory in both normal aging and AD, we examined whether the APOE e4 allele affected cognition, especially episodic memory, equally in persons with subjective cognitive decline (SCD), mild cognitive impairment (MCI), and dementia due to AD.

We further explored the relationship between cognition and APOE genotypes in patients with a diagnosis of mixed VaD/AD.

We hypothesized that people with the APOE e4, either heterozygote or homozygote, would have a different relationship with cognitive performance than non-e4 carriers would, regardless of SCD, MCI, and AD diagnoses, whereas no hypothesis is offered for the group with mixed VaD/AD. We further hypothesized that test scores would be worse in the dementia groups than in the SCD and MCI groups.

## Materials and Methods

### Norwegian Register of Persons Assessed for Cognitive Symptoms

Our study population came from the Norwegian Register of Persons Assessed for Cognitive Symptoms (NorCog), included and diagnosed during the period 2009–2018. NorCog is a national research and quality register for persons referred to the specialist health care service for assessment of memory impairment and possible dementia. The register is consent-based and has existed since 2009; 42 outpatient clinics currently collect these data from regular patients. NorCog is licensed by the Norwegian Data Inspectorate until 2030. Of those patients asked to give consent, 90% have answered positively and signed a consent form. The data collected in the register include a wide battery of neuropsychological tests, a comprehensive physical examination, blood-sample collection for various types of analyses, and cerebral imaging with magnetic resonance imagery or computed tomography (Braekhus et al., 2011). In addition, and in accordance with the Norwegian national guidelines regarding dementia, fluorodeoxyglucose-positron emission tomography (FDG-PET) and examination of concentration of amyloid-beta and tau protein in cerebrospinal fluid are increasingly performed in many patients with an uncertain diagnosis.

### Diagnoses

The dementia diagnoses recorded in NorCog are based on the International Classification of Diseases (ICD-10) criteria for research (World Health Organization [WHO], 1993) and made by experienced neurologists, geriatricians, or geriatric psychiatrists, usually in consensus meetings. The Winblad criteria are used for MCI (Winblad et al., 2004), and patients who complain about cognitive decline but show no cognitive impairment are given the diagnosis of SCD, in accordance with the Jessen criteria (Jessen et al., 2014). Patients diagnosed with SCD, MCI, AD, VaD and mix VaD/AD were included in the analyses (VaD and the mixed VaD/AD groups were combined). Patients with dementia due to Parkinson’s and Lewy body disease, Frontotemporal dementia (FTD) and unspecific dementia or dementia due to rare causes were excluded because of small samples with ApoE information. The patients are newly diagnosed in connection with their first assessments at an out-patient clinic in specialist health care for the elderly. The diagnoses were based on clinical examinations, including neuropsychological testing and MRI or CT of the brain. In additions, depending on clinical indication, extended neuropsychological tests were performed, CSF was analyzed (beta amyloid and tau protein), or FDG-PET was carried out. If AD was diagnosed, patients were offered cholinesterase inhibitor treatment immediately at the time of diagnosis.

### Study Population

The initial NorCog population comprised 10,457 patients, of which APOE information was recorded for 2,549 patients from five clinics. (No other clinics had APOE genotyped patients.) Of the 2,549 patients, we included 1,991 [Aker hospital (*n* = 173), Innlandet hospital (*n* = 231), Ullevål hospital (*n* = 1,017), Haugesund hospital (*n* = 80), and St. Olavs hospital (*n* = 490)] with a diagnosis of SCD, MCI, AD or mixed VaD/AD. Patients with other dementia diagnosis and APOE information were excluded because of the small number of patients in each diagnostic group.

### The Cognitive Test

The following cognitive tests were applied: The Mini Mental state examination (MMSE) (Folstein et al., 1975), the Consortium to Establish a Registry for Alzheimer’s Disease Word List (CERAD-WL) (immediate and delayed recall) (Morris et al., 1988; Hankee et al., 2016) and the Trail Making Test Part A (TMTA). MMSE is a well-known screening test for cognition that includes items measuring orientation, attention, memory, language, and visual-spatial skills. It includes 11 questions and takes 7–10 min to administer. Scores range from 0 to 30, with a higher score indicating better performance. A cut of between 24/23 is often used as a healthy/dementia indicator. The CERAD-WL test consists of 10 words presented over three learning trials, and a delayed recall trial presented 10 min after trial 3 administration. The maximum score for the learning trials is 30, and for delayed recall 10, with a higher score indicating better performance. Here normative data were available both from Norwegian and other populations (Morris et al., 1988; Spreen and Strauss, 1991; Kirsebom et al., 2019). The TMTA consists of 25 encircled numbers randomly arranged on a page; the participant is asked to draw a line through the circles in proper numerical order (Strauss et al., 2006). We recorded the time required in seconds to perform the test without mistakes, with a shorter time denoting better performance. There are many different norms developed related to the Trail Making test, most of these are summarized in Strauss et al. (2006).

The tests were administered by a trained nurse, a physician or psychometrician. The raw scores of the tests were used in the analyzes.

### APOE-Genotyping

Genotyping was performed using real-time PCR with allele-specific fluorescence energy transfer probes and melting curve analyses on a LightCycler 480 (Roche, Mannheim, Germany), with sense (GAAGGCCTACAAATCGGAACTG) and antisense (GGCTGCCCATCTCCTCCATC) primers and detection probes (LC-red705- ACATGGAGGACGTGCGCGG-Phosphate; NM_000041.3 [APOE]: c.388T > C variant nucleotide underlined) for the ε4 allele with the corresponding anchor probe (CTGCAGGCGGCGCAGGCCCGGCTGGGCGC-fluorescein). The 20 μL PCR reaction mix consisted of 1x LightCycler 480 Probes Master, 0.1 μM of sense and 0.5 μM of antisense primers, 0.14 μM of each probe, 10% dimethyl sulfoxide, and 5 μL of diluted DNA eluate (10–100 ng). The PCR touchdown protocol consisted of denaturation of DNA and activation of the polymerase (95°C, 5 min); 40 cycles of denaturation (95°C, 10 s), annealing (63°C stepping down 0.4°C/cycle to 59°C, 10 s), elongation (72°C, 10 s); denaturation and polymerase inactivation (99°C, 10 min); and melting curve analysis [38°C (1 min) to 77°C (ramp rate 1°C/s)]. The ε4 allele (rs429358) was identified by melting temperature 64°C vs. 56°C for wild type. The laboratory participates in an external quality assurance program (Equalis, Uppsala, Sweden) that includes APOE genotyping.

The number of years of education was self-reported. Anti-dementia medicine use was based on self-reports and coded using the ATC classification, as a binary variable with value 1 for those using medicines classified as N06D, and 0 for those not using one of these medicines.

### Statistical Modeling

To investigate cognitive performance by APOE genotype, the APOE genotypes were regressed against cognitive function (i.e., raw scores from the different tests) using linear regression, adjusting for age, sex, diagnosis, and education. The interaction between APOE and diagnosis was included to investigate whether cognitive function by APOE genotype differed by diagnosis. Predictions of mean cognitive scores, with 95% confidence intervals (CIs), from the regression models were performed using the margins commando in Stata with fixed values for the adjustment covariates (age 75 years, men and women weighted equally, 12 years of education). Furthermore, to investigate whether cognitive scores differed among the three APOE types, the monozygotic e4 was set as reference and the difference in cognition for those without the e4 and those with two e4s was predicted (with 95% CIs). Analyses were also performed without an interaction between APOE and diagnosis, in the full sample, in the sample restricted to those with a dementia diagnosis, and in a sample of those with the milder cognitive diagnoses.

### Ethics Statement

The study was reviewed and approved by the study Regional Ethics Committee for Medical and Biological Research (REK: 2019/316), and all participants gave written informed consent to participate in this study.

## Results

In our study population, 44% had one e4 allele and 12% had two. The occurrence of e4 differed by diagnosis and was most prevalent among AD patients (51% monozygotic and 20% homozygote) and least prevalent among SCD (38% monozygotic, 3% homozygote) and MCI patients (39% monozygotic and 12% homozygote) (see Table 1). SCD and MCI patients were younger than patients with a dementia diagnosis (see full APOE genotype distribution in Supplementary Table 1). Anti-dementia medicine use was used by 49 of the 1,991 patients (2.5%), mostly among those with AD (*n* = 37). The low number was mainly due the fact that the dementia diagnosis was made after the basic assessment as described in this article.

**TABLE 1:** Descriptive statistics.

	Men	Women	Age and education		Apoe E status, *n* (%)		
Diagnosis	*n* (%)	*n* (%)	Mean age (SD)	Education, years (SD)	No E4	One E4	Two E4
SCD	202 (20)	223 (21)	64.1 (11.5)	13.4 (4.0)	251 (59)	161 (38)	13 (3)
MCI	358 (35)	301 (28)	69.7 (10.6)	12.3 (3.7)	327 (50)	255 (39)	77 (12)
AD	256 (25)	374 (35)	73.5 (9.0)	11.7 (3.7)	181 (29)	324 (51)	125 (20)
VaD/AD	144 (14)	133 (12)	77.8 (6.7)	10.8 (3.4)	121 (44)	131 (47)	25 (9)
Total	960 (100)	1,031 (100)	70.9 (10.8)	12.1 (3.8)	880 (44)	871 (44)	240 (12)

*N = 1,991.*

Adjusted for age, sex, and education, those with the milder cognitive diagnoses (SCD and MCI) performed significantly better than the dementia groups on all cognitive tests. With similar adjustments, AD patients had the significantly lowest scores for the CERAD-WL tests. For TMTA, however, AD patients scored significantly better than patients in the mix + VaD group. For MMSE, there was no significant difference in scores among the dementia subgroups.

In crude analyses, e4 carriers with a diagnosis of SCD, MCI, or AD had significantly poorer cognitive performance on the CERAD-WL delayed recall test (Table 2) compared to non-carriers. For e4 carriers with a diagnosis of MCI, this was the case also for the immediate recall test, and for e4 carriers with SCD on the MMSE.

**TABLE 2:** Cognitive scores by diagnosis and E4 status.

	No E4	One E4	Two E4
	
Diagnosis	CERAD immediate recall: mean (SD)		
SCD	19.2 (4.9)	18.0 (5.6)	17.3 (7.1)
MCI	15.2 (4.4)	14.4 (4.3)*	14.8 (4.0)
AD	10.5 (4.2)	10.0 (4.3)	10.2 (4.3)
VaD/AD	10.4 (3.7)	9.9 (4.5)	9.9 (4.7)

	**CERAD delayed recall: mean (SD)**		
	
SCD	5.8 (2.3)	5.0 (2.7)*	4.0 (3.6)
MCI	3.8 (2.5)	3.0 (2.1)*	2.8 (2.2)*
AD	1.7 (1.8)	1.1 (1.4)*	0.8 (1.4)*
VaD/AD	1.6 (1.8)	1.3 (1.7)	1.2 (1.8)

	**MMSE: mean (SD)**		
	
SCD	28.0 (2.6)	27.3 (3.0)*	26.1 (3.5)
MCI	26.4 (3.1)	26.2 (3.0)	26.2 (2.8)
AD	21.9 (4.2)	21.5 (4.3)	21.5 (4.8)
VaD/AD	21.5 (4.2)	21.4 (3.9)	22.5 (4.2)

	**TMTA: mean (SD)**		
	
SCD	48.2 (37.8)	53.7 (45.0)	73.8 (79.2)
MCI	65.0 (35.7)	63.1 (32.3)	60.8 (28.6)
AD	96.3 (58.4)	91.0 (58.8)	86.8 (53.2)
VaD/AD	111.2 (65.0)	118.4 (67.5)	112.7 (69.7)

*Different from the group with No E4, tested in linear regression, *p* < 0.05.

*Crude mean values (Standard deviations: SD).*

**FIGURE 1:**
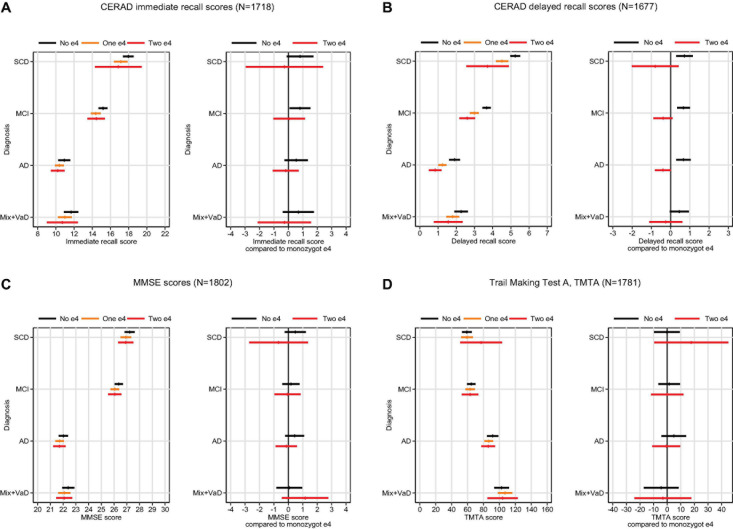
Mean cognitive scores on four cognitive tests **(A–D)** (with 95% CI bands) by e4 status and diagnosis (left panel), and predicted difference in cognitive scores for those without the e4 allele or homozygote e4 compared to those with monozygotic e4 (right panel).

In the analyses adjusted by age, sex, and education, allele type had the largest effect for cognitive performance assessed by the CERAD-WL delayed recall test, and less effect on the other tests—apart from immediate recall, in which the MCI patients without the e4 type scored significantly better than those with the e4-type (Figure 1). The interaction APOE by diagnosis was not significant (*p* = 0.99), however, suggesting an equal effect of APOE for all diagnoses.

Overall, for delayed recall, patients without the e4 allele scored 0.7 units better than did those with e4 allele(s) (*p* < 0.001). Furthermore, there was a significant inverse dose-response pattern between number of e4 alleles and cognitive performance; patients with one allele scored 0.4 units better than did those with two alleles (*p* = 0.006), and those without e4 scored 0.7 units better than did those with one e4 (*p* < 0.001). This pattern did not differ among the diagnostic groups. When diagnostic groups were analyzed individually, however, no significant difference between those with two and one e4 was found, probably due to lack of statistical power. A significant difference, however, was observed between patients with no e4 and those with one or two e4s (see Figure 1B, right panel).

## Discussion

The strongest association between APOE e4 and cognition was observed in the CERAD-WL delayed recall, and this association was similar across diagnoses (i.e., there was no APOE e4 ^∗^ diagnosis interaction). The finding concurs with the clinical hallmark of AD, a progressive reduction of episodic memory (McKhann et al., 2011). Nevertheless, this association was related not only to AD; it was found in all the diagnostic groups we examined. Overall, for the APOE e4, we also found a tendency for a dose-response effect on the delayed CERAD-WL delayed-recall test, with the weakest performance among patients within the homozygote e4 group. In diagnosis-specific analyses, however, this dose response did not reach statistical significance, likely due to low statistical power. Memory decline is also seen in healthy e4 carriers compared to non-carriers (Caselli et al., 2009). In children it is reported that e4 carriers have smaller entorhinal cortexes compared to non-carriers, which could possibly be interpreted as a developmental effect (Shaw et al., 2007). One will rarely see this effect on cognition in children, and it has been suggested that memory impairment becomes manifest after age 64 (Rawle et al., 2018). Homozygote carriers will present with most memory impairment (Rawle et al., 2018). Thus, it is possible that the observed reduction of episodic memory among e4 carriers is due not only to AD in a preclinical (MCI) or dementia stage but could just as well be a genetic cause of reduced development of the hippocampal area and thus be related to reduced memory performance that will occur in late life. Our findings could be interpreted in line with such an assumption or theory. Further findings could support our assumption/theory: Only scores on the CERAD-WL (especially delayed recall) were associated with the APO E e4 status across diagnoses. We saw no associations between e4 genotype and processing speed, scores on the MMSE, or TMTA. Therefore, the other cognitive deficits may not be related to the e4 allele, or they may be secondary to poor episodic memory.

Comparing the dementia groups, no differences were found among the AD and AD/VaD groups on the MMSE. On the TMTA, the AD group performed better than the AD/VaD group. Thus, the memory effect of the e4 allele does not seem to be specific to AD, because the same effect is seen in the diagnostic groups of SCD and MCI. There is a higher prevalence of the APOE-e4 allele is the AD group, however, and performance impairment on the CERAD-WL is larger in the AD than in any of the other diagnostic groups, especially for delayed recall. The large cognitive deficits on the delayed word list recall in AD may therefore be attributed to factors other than the APOE e4 allele. Nevertheless, the allele seems to be at least partly responsible for the memory problems related to word list recall. Looking at our data, and in line with Caselli et al. (2009) and Rawle et al. (2018) we suggest that the e4 allele probably influence the aging process of the brain. Those with the e4 allele as a group may have fewer brain reserves as part of the aging process. Not everyone with e4 allele will develop dementia, however, which probably indicates great variation. To have the e4 allele is probably neither sufficient nor necessary for developing dementia—neither AD nor VaD, even though it is a strong risk factor. The gene may contribute to the memory difficulties, however, especially delayed recall.

### Limitations

This study has some limitations. First, its cross-sectional design makes it impossible to observe who will develop dementia belonging to the SCD and MCI diagnostic groups. Some of these patients may have AD in an early, preclinical stage. Second, we have not included neuropsychological tests that cover most cognitive domains, and thus cannot be sure that APOE genotype may be associated with other cognitive domains in addition to memory. A strength of the study is the large sample with patients who have been examined comprehensively and diagnosed with standardized criteria.

### Conclusion

Our interpretation of the data is that the APOE e4 allele may contribute to the development of dementia, but the association between the e4 allele and the delayed recall test is also seen in the SCD and MCI groups. However, this is a cross-sectional study and the data needs to be confirmed in other kinds of studies. We, nevertheless, suggest that the effects of e4 allele can make the brain more vulnerable to dysfunction, which, in turn, could reduce brain reserves and contribute to the development of dementia.

## Data Availability Statement

The data analyzed in this study was subjected to the following licenses/restrictions: The data was collected from the Norwegian Register of Persons Assessed for Cognitive Symptoms (NorCog). The data can be available after approvement from the board of the database. Requests to access these datasets should be directed to Marit Nåvik, naam@sthf.no.

## Ethics Statement

The studies involving human participants were reviewed and approved by the Regional Ethics Committee for Medical and Biological Research (REK: 2019/316) and all participants gave written informed consent. The patients/participants provided their written informed consent to participate in this study.

## Author Contributions

KH initiated and planned the study. BS, PH, and KE took part in the planning of the study. BS did all the statistical analyzes. KH and BS wrote the first draft of the manuscript. All authors took part in the writing process.

## Conflict of Interest

The authors declare that the research was conducted in the absence of any commercial or financial relationships that could be construed as a potential conflict of interest.
