# Supplementary material for: Cognition in Patients With Memory Difficulties and Dementia Relative to APOE e4 Status
Source: Front Psychol. 2021 Jun 14;12:686036. doi: 10.3389/fpsyg.2021.686036 (PMC8236580; doi:10.3389/fpsyg.2021.686036)
Supplement: Supplementary file 1 [file Table_1.docx]

**Appendix**

Supplementary Table 1. APOE genotype distribution (%) by diagnosis

| APOE | SCD | MCI | AD | VaD/AD | Total |
| --- | --- | --- | --- | --- | --- |
| E2/E2 | - | - | - | - | 0.4 |
| E2/E3 | 9.4 | 7.3 | 3.8 | 2.5 | 6.0 |
| E2/E4 | 4.0 | 2.6 | 3.0 | 2.5 | 3.0 |
| E3/E3 | 49.7 | 41.6 | 24.8 | 40.8 | 37.9 |
| E3/E4 | 33.9 | 36.1 | 48.4 | 44.8 | 40.7 |
| E4/E4 | 3.1 | 11.7 | 19.8 | 9.0 | 12.1 |
| Total | 100 | 100 | 100 | 100 | 100 |

*E2/E2 type has <5 for individual diagnoses and therefore not reported for individual diagnoses.
